# IL-33/ST2 antagonizes STING signal transduction via autophagy in response to acetaminophen-mediated toxicological immunity

**DOI:** 10.1186/s12964-023-01114-3

**Published:** 2023-04-20

**Authors:** Zengbin Wang, Pei Sun, Banglun Pan, Jiacheng Qiu, Xiaoxia Zhang, Shuling Shen, Xiaoling Ke, Nanhong Tang

**Affiliations:** 1 Department of Hepatobiliary Surgery and Fujian Institute of Hepatobiliary Surgery, Cancer Center of Fujian Medical University, Fujian Medical University Union Hospital, Fuzhou, China; 2 College of Clinical Medicine for Obstetrics & Gynecology and Pediatrics, Fujian Medical University, Fuzhou, China; 3 Key Laboratory of Ministry of Education for Gastrointestinal Cancer, Research Center for Molecular Medicine, Fujian Medical University, Fuzhou, China

**Keywords:** IL-33/ST2, Acetaminophen-induced liver injury, STING, Autophagy, Acetylation

## Abstract

**Background:**

Interleukin-33 (IL-33), defined as "alarming", exert diverse functions through signaling via the suppression of tumorigenicity 2 (ST2). However, the physiological roles of IL-33/ST2 signaling during acetaminophen (APAP)-induced liver injury are still poorly understood by modern medicine (AILI). This research aims to explore the relationship between IL-33/ST2 and stimulator of interferon (IFN) response cGAMP interactor 1 (STING)-mediated signal transduction.

**Methods:**

C57BL/6N mice (WT) and IL-33-deficient mice (KO) were intraperitoneally injected with APAP (250 mg/kg). Recombinant IL-33 (500 ng/mouse) and the cGAS/STING inhibitor RU.521 (200 g/kg) were combined to treat AILI. For mechanistic research in vitro, CRISPR-mediated KD technology, immunoprecipitation, mass spectrometry, and immunofluorescence were utilized.

**Results:**

We discovered that IL-33 deficient mice had increased APAP-induced hepatotoxicity, DNA accumulation, and type 1 IFN production. Mechanistic analysis revealed that IL-33/ST2 enhanced the interaction between Beclin-1 and STING, disrupting STING dimerization, IRF3 phosphorylation, nuclear transport, and IFN-1 gene transcription in HepaRG and Huh7 cells. Beclin-1 interacted with the C-terminus of STING, causing Lys338 acetylation and autophagy degradation of STING. ST2 depletion increased STING signal transduction and IFN-1 promoter activity. Surprisingly, the cGAS/STING inhibitor RU.521 and recombinant IL-33 together improved AILI in vivo.

**Conclusions:**

These results shed insight on the potential of inhibiting cGAS/STING as a therapy for AILI and emphasize the crucial role of IL-33/ST2 signaling in the regulation of APAP-induced STING signaling.

Video Abstract

**Graphical Abstract:**

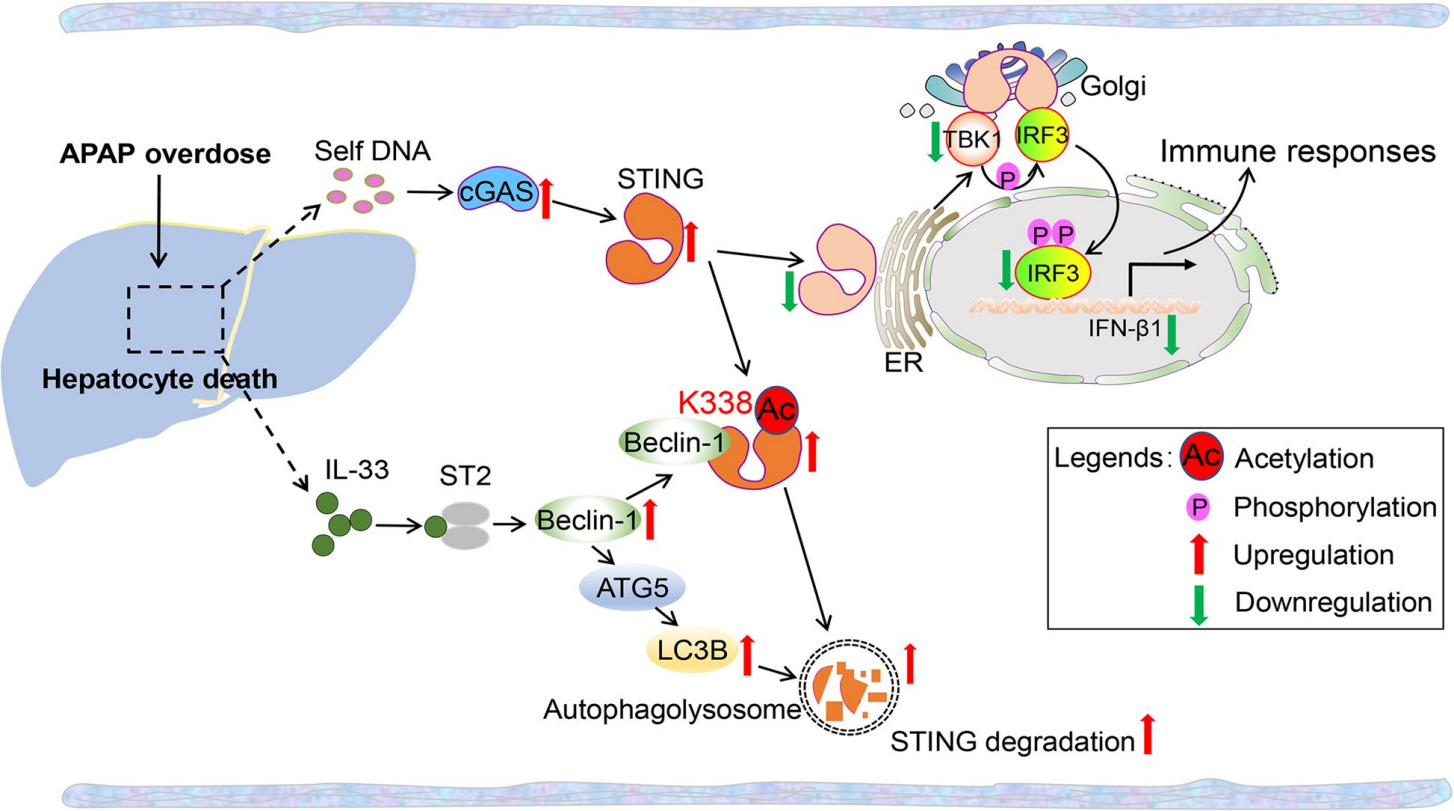

**Supplementary Information:**

The online version contains supplementary material available at 10.1186/s12964-023-01114-3.

## Background

Acetaminophen (N-acetyl-p-aminophenol, APAP), is an antipyretic and analgesic, which is mostly used worldwide as analgesics and antipyretics. However, APAP overdose gives rise to severe acute liver injury (ALI) and even acute liver failure (ALF) [1]. It is well known that APAP-induced toxicity is related to N-acetyl-p-benzoquinone imine (NAPQI) [2], a cytotoxic metabolite, generated primarily under the action of CYP-dependent cytochrome P450 [3]. Currently, N-acetylcysteine (NAC) is the only drug for APAP-induced liver injury, but it is only effective in the early stage of ALI [4]. Although there are potential new drugs used in ALI treatment, such as the re-purposed drug fomepizole (4-methyl pyrazole) and the new entity calmangafodipir, both drugs require controlled phase III trials [5]. Thus, it is urgent to discover novel and effective therapies to attenuate APAP overdose-induced ALI.

Recent studies have found that APAP-induced ALI is associated with the liver immune microenvironment. As an example, APAP overdose caused aberrant innate cell (Kupffer cells and neutrophils) infiltration and activation, and higher levels of pro-inflammatory TNF-α and IL-6, but lower levels of anti-inflammatory IL-10 [6]; IL-1α was predominantly secreted by Kupffer cells in response to damage-associated molecular patterns (DAMPs) released from necrotic hepatocytes during AILI [7]. IL-33, a member of the IL-1 family, first discovered by Schmitz et al.[8], interacts with ST2 [9]. It is thought to be released and function as an alarm during cellular stress or death. Emerging evidence indicates that IL-33 acts as a “dual function cytokine”, exerting different roles in the regulation of transcriptional effects and inflammatory response [10]. Recently, evidence suggests that APAP induces the generation of IL-33 [11]. We previously have shown that IL-33 can affect APAP-induced liver injury by mediating autophagy [12]. Due to the importance of immune factors, multiple cell phenotypes and signaling pathways are closely associated with IL-33. More work is required to investigate the mechanism of IL-33 against APAP-induced liver injury.

In the present study, we elucidate a novel insight that IL-33/ST2 signaling affected APAP-induced liver necrosis and type 1 IFN-mediated innate immune response during APAP-induced injury. Targeting STING signaling may hold further truthful research avenues for pharmacological interventions during drug-induced acute liver injury.

## Materials and methods

### Antibodies and reagents

Antibodies and reagents were obtained from the respective manufacturers: RP-MI-1640 medium (HyClone, South Logan, UT), fetal bovine serum (Gemini Bio, Sacramento, CA), penicillin–streptomycin (Solarbio, Beijing, China), Fluoromount with 4’, 6-diamidino-2-phenylindole (DAPI) (Sigma-Aldrich, St. Louis, MO), Dulbecco’s Modified Eagle Medium (Gibco, Grand Island, NY), Acetaminophen (APAP) (Yuanye Bio-Tech Co., Shanghai, China), Lipofectamine 3000 (Invitrogen, Carlsbad, CA), Recombinant human IL-33 and mouse IL-33 (rhIL-33, Elabsceience Bio., Wuhan, China), puromycin (Solarbio), Sytox Green (Invitrogen, Carlsbad, CA), anti-Flag (ABMART, Shanghai, China), anti-Myc (ABMART), anti-HA (ABMART), anti-tubulin (ABMART), anti-LC3B (Cell Signaling Technology, Beverly, MA), anti-Beclin-1 (Cell Signaling Technology), anti-IRF3 (Cell Signaling Technology), anti-p-IRF3 (Cell Signaling Technology), anti-STING (Cell Signaling Technology), anti-ST2 (Proteintech, Wuhan, China), anti-LaminB (Proteintech, Wuhan, China), Protein A/G PLUS Agarose (ABMART). cGAS/STING inhibitor RU.521 (Synonyms: RU320521, CAS 2262452–06-0), 3-Methyladenine (3-MA) (CAS 5142–23-4), and MG132 (CAS 133407–82-6) were purchased from Merck, Darmstadt, Germany.

### Animal model and in vivo studies

The male C57BL/6N mice (WT), weight approximately 18–22 g, six to eight-week-old, were obtained from Shanghai SLAC Laboratory Animal Co., Ltd. The IL-33-deficient mice (KO) in the C57BL/N genetic background were kindly provided by Dr. *Susumu Nakae* (Institute of Medical Science, University of Tokyo, Japan). Our previous studies have verified the genotyping of mice [12]. All mice were maintained under specific-pathogen-free (SPF) conditions in a controlled environment of 21–23 °C with 40–55% humidity. Mice were fasted overnight but had free access to water before the experiments. All studies were performed according to the institutional protocols approved by the Experimental Animal Ethics Committee of Fujian Medical University. The APAP-induced liver injury model was prepared as described previously [12]. In brief, APAP was dissolved in warm saline before gavage. Mice fasted for 12 h before APAP administration (250 mg/kg) or warm sterile saline as vehicle intraperitoneally injection. To assess the effect of cGAS/STING inhibitor RU.521, mice were injected with RU.521 (200 μg/kg) and rmIL-33 (500 ng/mouse) before APAP administration (250 mg/kg). The serum alanine aminotransferase (ALT), aspartate aminotransferase (AST) and GSH levels were performed using an assay kit (Jiancheng Bioengineering Institute, Nanjing, China).

### Cell culture

The HepaRG cells were obtained from Millipore (Cat# C103485, RRID: CVCL_9720) maintained in RPMI 1640. The HEK293T cells (ATCC Cat# CRL-3216, RRID: CVCL_0063) and Huh7 cells (JCRB Cat# JCRB0403, RRID: CVCL_0336) were maintained in Dulbecco’s Modified Eagle Medium (DMEM). All cells were supplemented with 10% fetal bovine serum (Gibco) and 100 U/mL Penicillin/Streptomycin at 37 °C in a 5% CO_2_ atmosphere.

### Plasmid constructs and transfection

GFP-LC3 plasmid was kindly provided by Dr. *Gutterman* (University of Texas MD Anderson Cancer Center, Houston, TX) which has been described elsewhere [13]. HA-Ub, HA-STING^1−190^, HA-STING^191−379^, GFP-Golgi and Flag-Beclin-1 were obtained from Tsingke Biotechnology Co., Ltd. pCMV-C-HA, pCMV-C-Flag, pCMV-C-YFP, pCMV-C-Myc, pCMV-C-mCherry, and pCMV-C-EGFP were obtained from Beyotime. cDNA fragments prepared from HepaRG cells, amplified by PCR from cDNA, encoding human Beclin-1, ST2, STING, IRF3, TBK1 and cGAS were constructed and fused with Myc-, HA-, Flag-, YFP-, mCherry- or GFP, respectively, according to the manufacturer’s instructions. The plasmids pCMV-HA-STING, pCMV-Flag-STING, pCMV-Myc-STING, pCMV-mCherry-STING, pCMV-HA-TBK1, pCMV-Flag-TBK1, pCMV-Myc-ST2, pCMV-Flag-ST2, pCMV-Flag-Beclin-1, pCMV-EGFP-Beclin-1, pCMV-YFP-IRF3, pCMV-HA-cGAS were constructed for this study. The primers used were given in Table S1. Plasmids for human IL-33-specific shRNAs (shIL-33), ST2-specific shRNAs (shST2) and shScram (containing a scrambled non-targeting sequence) were obtained from GenaPharma (Shanghai, China).

To establish HepaRG/shIL-33, HepaRG/shST2 and Huh7/shST2 cells, HepaRG and Huh7 cells were transfected with plasmids using Lipofectamine 3000 and selected by puromycin according to the manufacturer’s instructions. To generate expression vectors pCDH-ST2 (ST2-OE), the coding sequences of the human ST2 gene were cloned into the NheI and BamHI sites of the control plasmids pCDH-CMV-MCS-EF1-copGFP vector (System Biosciences, CA, USA). All constructs were verified by sequencing. The forward primer contained a NheI site 5′-CTAGCTAGCATGATTGACAGACAGAGAATGGGAC-3′, and the reverse primer the BamH1 site 5′-CGGGATCCCCAAAGTGTTTCAGGTCTAAGCATGC-3′. To build HepaRG/ST2-OE and Huh7/ST2-OE cells, HEK293T cells were co-transfected with the lentivirus expression vector (pSPAX2, and pMD2G) and pCDH-ST2 using Lipofectamine 3000. After 48 h, the lentivirus supernatant was harvested to infect HepaRG and Huh7 cells. Transduced HepaRG cells were selected by puromycin (3 μg/mL) for 3 days.

Knockdown of the human ATG5 gene (ATG5 KD) using CRISPR/Cas9 technology. The guide RNA (gRNA) targeting the ATG5 was obtained from Tsingke Biotechnology. To generate the stable ATG5 knockdown cells, we ligated gRNA to the pX459 plasmid to obtain CRISPR/Cas9-gRNA plasmid, then the plasmid was transfected into HepaRG and Huh7 cells. The single-cloned cells were selected for expansion and culture. The efficiency of knockdown was confirmed by immunoblotting.

### Dual-luciferase reporter assay

The human IFN-β1 promoter was obtained from the NCBI and cloned into the pGL4.10 vector (Promega, Madison, USA). The sequences of the plasmids were verified using sequencing. HepaRG and Huh7 cells co-transfected with HA-tagged STING, pGL4-IFN-β1 promoter-Luc reporter, or pGL4.10-basic control vectors for 24 h, were subjected to pre-incubation with or without rhIL-33 (100 ng/mL) for 2 h and then treated with 3-MA (10 mM) for 12 h, or proteasome inhibitor MG132 (10 μM) for 12 h. pRL-TK (Promega) was used as an internal control. Cell lysates were harvested for the dual-luciferase assay, which was performed according to the manufacturer's recommendations (Promega).

### Immunoprecipitation and immunoblotting

In brief, cells were lysed in RIPA buffer (50 mM Tris–HCl, pH 7.4; 150 mM NaCl; 1 mM EDTA; 1% NP-40; 0.25% NaDoc; 10% Glycerol) containing phenylmethylsulfonyl fluoride (PMSF; Solarbio, P8340) and protease inhibitor cocktail (1:100, P8340; Sigma-Aldrich) for 15 min at 4 °C. 5% of total lysates were used as input for each sample. The remaining lysate was incubated with 1 μg of primary antibody on the rotator at 4 °C overnight. Protein G sepharose was then added and incubated for another 4 h at 4 °C. Protein G sepharose-enriched complexes were resolved on SDS-PAGE gels and transferred onto PVDF membranes.

Immunoblotting of the cell lysates and immunoprecipitates was performed using primary antibodies as indicated overnight at 4 °C and then with secondary antibodies for 1 h at room temperature. The bands were detected and visualized using a Hypersensitive ECL Chemiluminescence Kit (ABP Biosciences, Beltsville, MD).

### Mass spectrometry

HA-tagged STING was transfected into HepaRG cells. At 24 h post-transfection, cells were treated with rhIL-33 (100 ng/mL) for 12 h. HA-STING protein precipitated by Beclin-1 was purified and then analyzed using mass spectrometry. Mass spectrometry was provided by Shanghai Luming Biological Technology co.Ltd.

### Nuclear and cytoplasmic extraction

Nuclear and cytoplasmic extraction was conducted with the Nuclear and Cytoplasmic Protein Extraction Kit (Beyotime, Wuhan, China) according to the manufacturer’s instructions. The extracted fractions were used for immunoblotting analysis.

### RNA extraction and real-time PCR analysis

Total RNA was extracted by Trizol reagent (Thermo Fisher, Waltham, MA) following the manufacturer’s instructions. Total RNA (2 µg) was used to perform reverse transcription with Revert Aid First Strand cDNA Synthesis Kit (Thermo Fisher). Quantitative real-time RT-PCR analysis was performed with a Quantstudio3 instrument (Thermo Fisher) using Fast Start Universal SYBR Green Mix (Roche, Mannheim, Germany). The primers, synthesized by Sangon Biotech., Shanghai, China., and used for real-time RT-PCR were displayed in Table S2. The method of 2^−∆∆Ct^ was used to calculate the relative mRNA level. Data were normalized to GAPDH expression.

### Enzyme-Linked Immunosorbent Assay (ELISA)

Supernatants from mouse vessels were collected. Briefly, serum was separated by centrifugation at 4000 rpm for 10 min at various time points after APAP injection. Serum IL-33 levels were measured using commercially available ELISA kits (Elabsceience Bio.) following the manufacturer’s instructions.

### Statistical analysis

All experiments were performed at least three times independently. GraphPad Prism 7 software (San Diego, CA) was used for statistical analyses. The results were presented as mean ± SD. Statistically significant differences were determined by Student's unpaired t‑tests or one-way analysis of variance (ANOVA). Survival curves were compared using the log-rank (Mantel-Cox) test. A *p*-value < 0.05 was considered statistically significant.

## Results

### IL-33-deficient mice aggravate APAP-induced hepatotoxicity, massive DNA accumulation, and type 1 IFN production

**Fig. 1:**
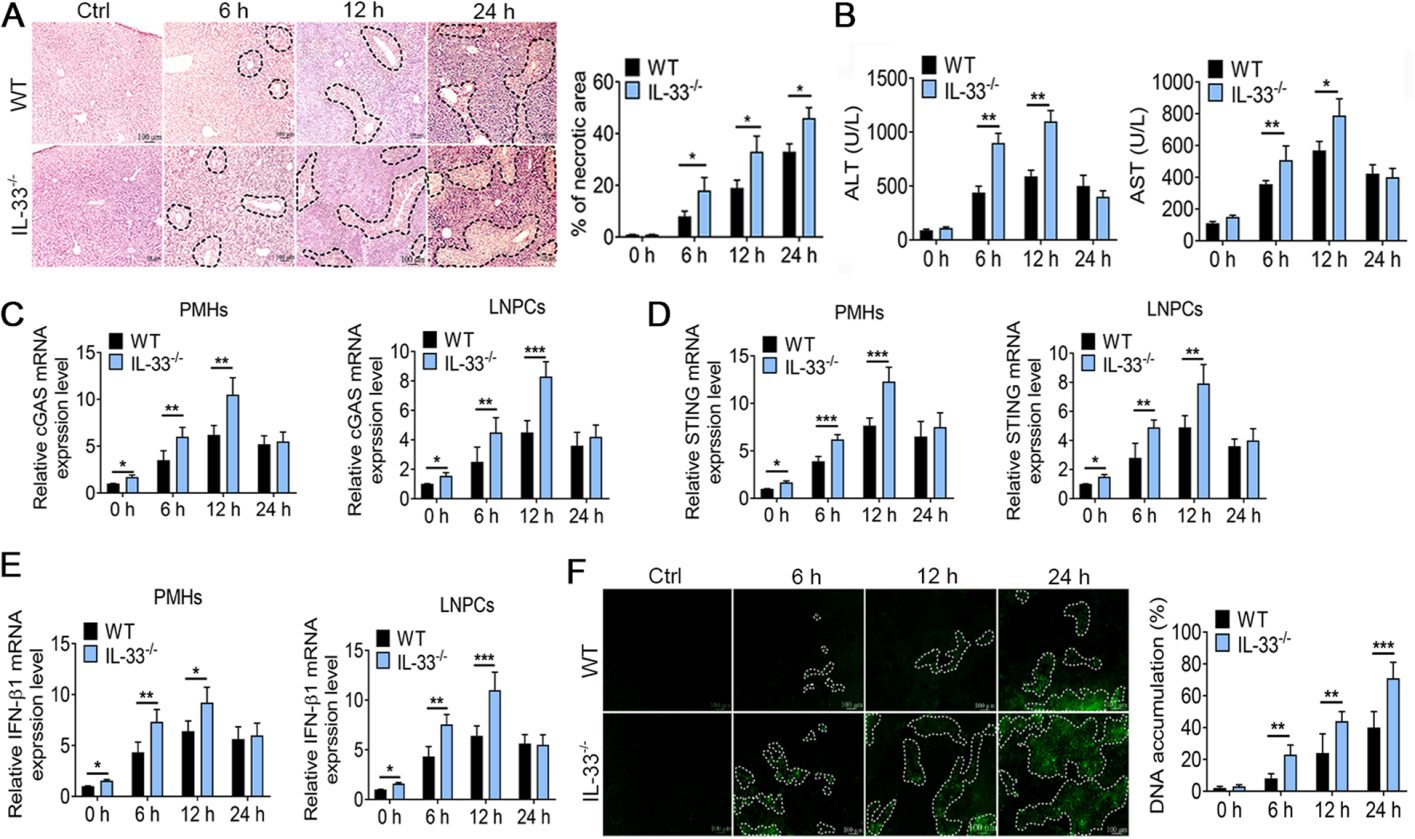
IL-33-deficient mice have massive DNA accumulation and type 1 IFN production during APAP-induced injury. **A** H&E staining was used to observe hepatic necrosis at different times after APAP (250 mg/kg) administration (*n* = 6), scale bar = 100 µm. **B** Serum ALT/AST was detected in WT and IL-33.^−/−^ mice at 6, 12, and 24 h after APAP (250 mg/kg) administration injection (*n* = 6). **C**-**E** The mRNA levels of cGAS, STING and IFN-β1 were measured by qRT-PCR after 6 h, 12 h and 24 h APAP (250 mg/kg) treatment (*n* = 6). **F** Confocal microscopy showing DNA accumulation in the liver following APAP challenge (Sytox green staining; *n* = 3/group), scale bar = 100 µm. *P < 0.05, **P < 0.01, ***P < 0.001

Some studies testified that an overdose of the APAP resulted in massive hepatocyte necrosis, resulting in a range of DAMPs [14]. Pedro Elias Marques et al. also found that administration of APAP in vivo caused massive DNA deposition within liver necrotic areas [15], driving inflammation through pattern recognition receptors (PRRs) in both immune and non-immune cells. These cells activate the immune system by expressing different DNA sensors including cyclic guanosine monophosphate-adenosine monophosphate synthase (cGAS), STING, and Toll-like receptor 9 (TLR9), which triggers the TANK binding kinase 1 (TBK1)-mediated activation of interferon regulatory factor IRF3 and production of type I IFNs [16]. We previously have shown that APAP induces the expression of IL-33 in liver cells [12]. Whether IL-33 participates in or affects DNA deposition and its inductive signal transduction induced by APAP has not been reported. To this end, we first established an APAP-induced liver injury mouse model (250 mg/kg APAP, intraperitoneal (i.p.)) in both wild-type (WT) and IL-33^−/−^ mice (KO). ELISA assay revealed a significant increase in serum IL-33 in WT mice after APAP administration at 6 h, 12 h and 24 h (Fig. S1). H&E staining demonstrated that IL-33^−/−^ mice showed great hepatic necrosis at 6 h – 24 h post-APAP treatment (Fig. 1A). Biochemical assays showed that the levels of serum alanine aminotransferase (ALT) and aspartate aminotransferase (AST) were higher in IL-33^−/−^ mice than in WT mice after APAP administration at 6 h, 12 h and 24 h (Fig. 1B). We further tested the cGAS and STING mRNA levels both in primary hepatocytes (PMHs) and liver nonparenchymal cells (LNPCs) by qRT-PCR. Interestingly, the relative mRNA levels of cGAS and STING in PMHs and LNPCs were higher in the early stage of liver injury and gradually decreased in the late stage. Moreover, both PMHs and LNPCs had higher levels of cGAS and STING in IL-33^−/−^ mice compared to WT mice (Fig. 1C and D). Since the DNA-sensing pathways triggered type 1 interferon response [17], we next investigated the transcription level of type 1 IFN (IFN-β1) production. Compared with WT mice, the relative mRNA level of IFN-β1 was also greatly upregulated in the early stage of liver injury in IL-33^−/−^ mice both in PMHs and LNPCs (Fig. 1E). Concomitantly, significant DNA deposition in extracellular around hepatocyte cell necrosis was observed by Sytox Green (Fig. 1F). These studies suggested that IL-33 deletion promoted DNA accumulation and cGAS/STING/IFN signaling activation.

### IL-33/ST2 potentiates the degradation of STING via Beclin-1-medicated autophagy

**Fig. 2:**
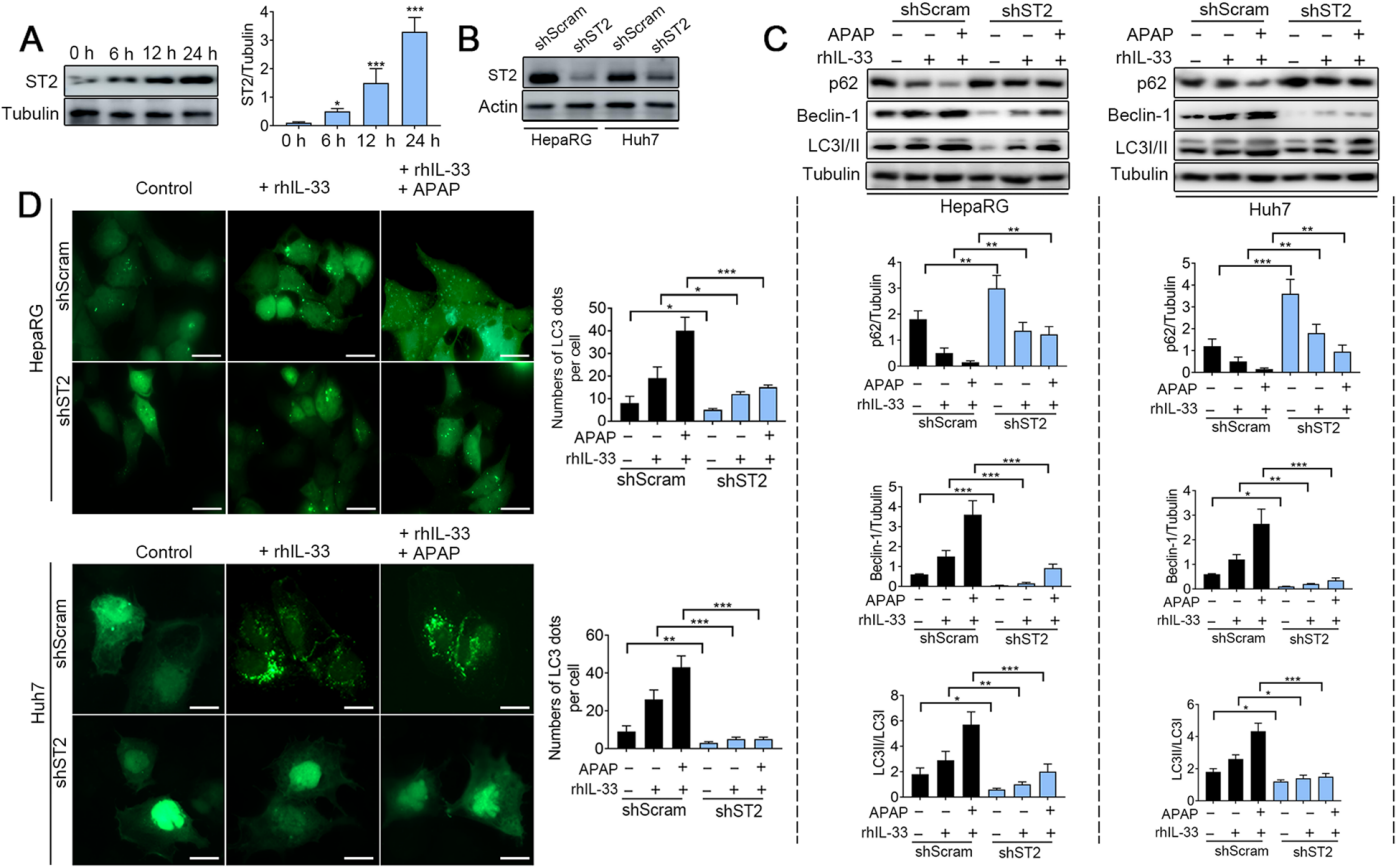
Knockdown of ST2 inhibits APAP-induced autophagy. **A** APAP (250 mg/kg) was used for intraperitoneal injection for the indicated time. Western blotting was used to detect the expression of ST2 in the liver with the indicated antibodies (*n* = 3). **B** We generated ST2 knockout HepaRG and Huh7 cells (*n* = 3). **C** Immunoblot analysis of Beclin-1, LC3I/LC3II, and tubulin in ST2 wild-type and knockout cells that were treated with APAP (10 mmol/L) for 12 h or rhIL-33 (100 ng/mL) for 12 h (*n* = 3). **D** ST2 wild-type and knockout cells were transfected with a plasmid expressing GFP-LC3 and stimulated with APAP (10 mmol/L) for 12 h or rhIL-33 (100 ng/mL) for 12 h. Images were captured by confocal microscopy. The numbers of GFP-LC3 dots per cell were quantified (n > 100 cells from three independent experiments). Scale bar: 10 µm. *P < 0.05, **P < 0.01, ***P < 0.001

Based on the above results, we speculated that IL-33 released from APAP-stimulated cells will bind with its receptor ST2 and trigger downstream signal transduction. Herein, we found that the expression of ST2 has greatly increased in the liver after APAP intraperitoneally injection in WT mice (Fig. 2A). Therefore, it is necessary to explore the relationship between IL-33/ST2 signaling and STING. HepaRG and Huh7 cells were next transfected to obtain the shST2 group and shScram group (Fig. 2B). Recombinant human IL-33 (rhIL-33) administration or co-stimulated by rhIL-33 and APAP strongly induced autophagy as determined by enhancing the expression of Beclin-1 and LC3-II, and the degradation of p62 (Fig. 2C, lanes 1–3), whereas ST2 knockdown reversing the effects (Fig. 2C, lanes 3 and 6). To test the formation of autophagosomes, we transfected a plasmid encoding microtubule-associated protein 1 light chain 3 betas (LC3B) fused with the green fluorescent protein. As can be seen in Fig. 2D, whether stimulated by rhIL-33 or co-stimulated by APAP and rhIL-33, ST2 knockdown cells had a low percentage of GFP-LC3 puncta compared with the shScram cells.

**Fig. 3:**
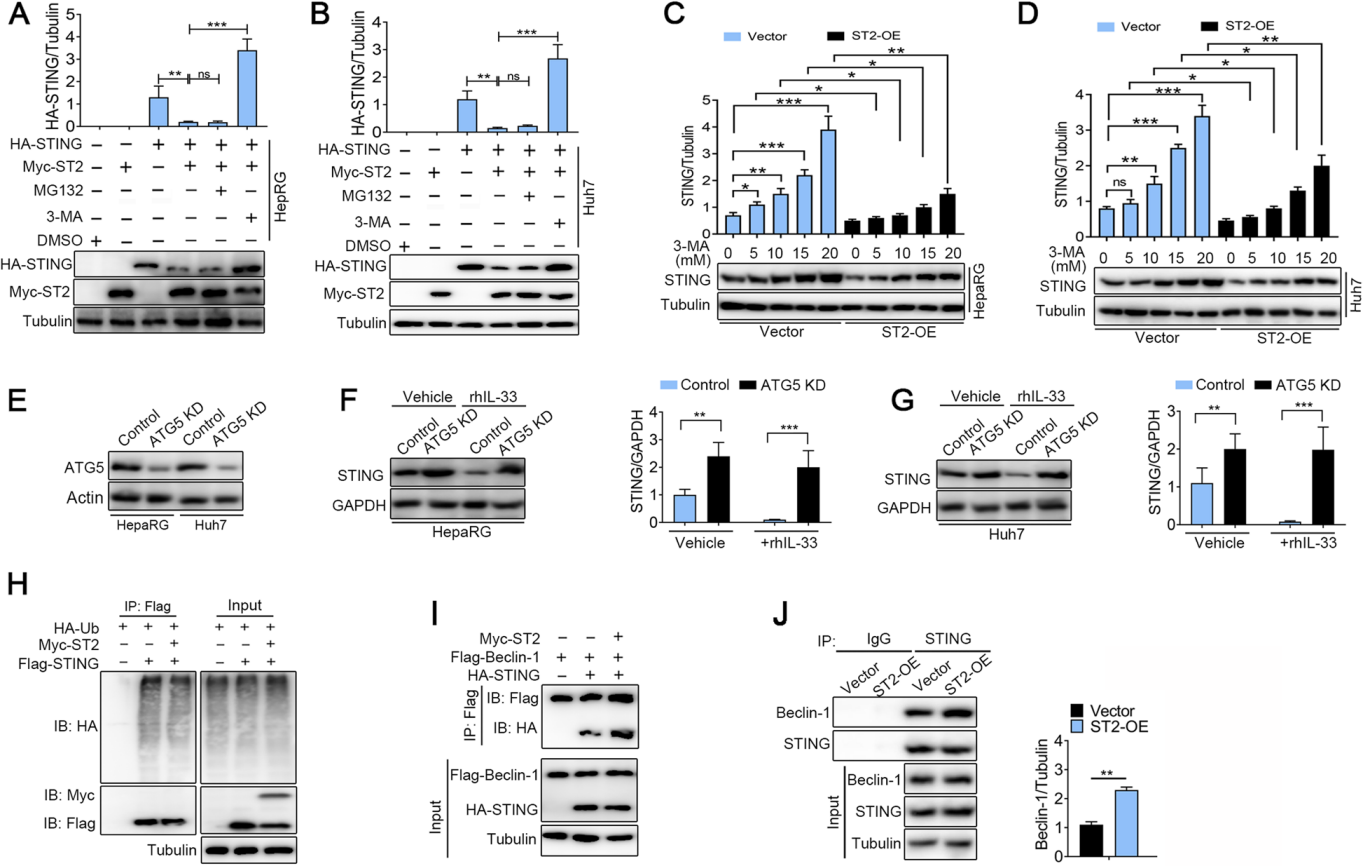
ST2 promotes Beclin-1 mediated autophagy degradation of STING rather than ubiquitin degradation in HepaRG and Huh7 cells. **A** and **B** HepaRG and Huh7 cells were transfected with Myc–tagged ST2 and HA-tagged STING plasmids for 24 h and then treated with 10 μM MG132 or 10 mM 3-MA for the indicated times (*n* = 3). The total cell lysates were then analyzed by western blot with the indicated antibodies. **C** and **D** Immunoblot analysis of STING protein level in HepaRG and Huh7 cells over-expressing ST2 after treatment with 3-MA (0, 5, 10, 15, 20 mM) for 12 h (*n* = 3). **E** ATG5 KD cells were constructed using CRISPR technology in HepaRG and Huh7 cells. Immunoblot analysis of STING protein level. **F** and **G** Immunoblot analysis of STING protein level in HepaRG and Huh7 cells after treatment with rhIL-33 for 12 h (*n* = 3). **H** Immunoprecipitation and immunoblot analysis of HepaRG cells transfected with plasmids encoding Myc-ST2, HA-Ubiquitin (HA-Ub), Flag-STING and empty vector for 24 h (*n* = 3). **I** Immunoprecipitation and immunoblot analysis of HepaRG transfected with plasmids encoding Myc-ST2, HA-STING, Flag-Beclin-1 an empty vector for 24 h (*n* = 3). **J** ST2-OE and vector cell lysates were immunoprecipitated using STING antibody and blotted with the indicated antibodies (*n* = 3). *P < 0.05, **P < 0.01, ***P < 0.001

What is the molecular mechanism underlying the effect of IL-33/ST2 on regulating STING? We hypothesized that IL-33/ST2 regulated STING through autophagy or proteasomal pathways. Firstly, we examined if IL-33/ST2 could affect the stability of the STING protein. HepaRG and Huh7 cells were transfected with Myc-tagged ST2 and HA-tagged STING for 24 h, then cells were treated with autophagy inhibitor 3-MA (10 mM) for 12 h or a proteasomal inhibitor MG132 (10 μM) for 12 h. Immunoblotting analysis showed that ST2-mediated degradation of STING was completely recovered by adding the 3-MA but not MG132 (Fig. 3A and B). We further analyzed changes in the STING protein levels in cells over-expressing ST2 (ST2-OE) in the presence of 3-MA. No matter in the control group or the overexpression ST2 group, 3-MA treatment affected STING protein expression (Fig. 3C and D). These results suggested that IL-33/ST2 regulated STING through autophagy pathways.

To verify the above results, we constructed ATG5 KD cells using CRISPR technology both in HepaRG and Huh7 cells (Fig. 3E). ATG5 depletion was performed to inhibit the autophagy process [18]. ATG5 KD increased the level of STING protein expression with or without rhIL-33 treatment (Fig. 3F and G, lanes 1 and 2, lanes 3 and 4). Furthermore, we found that ST2 did not catalyze the ubiquitination of STING and downregulate the protein levels of STING (Fig. 3H). Inversely, we found an interesting phenomenon that ST2 promoted Beclin-1, an autophagy-initiating protein, binding to STING (Fig. 3I), indicating a degradative role of ST2 for STING on autophagy. To further confirm this effect, we measured the level of Beclin-1 protein precipitated by STING using ST2-OE and ST2-vector cells’ lysates. As shown in Fig. 3J, the level of Beclin-1 protein precipitated by STING was significantly enhanced in ST2-OE cells compared with vector cells. These data suggested that IL-33/ST2 promoted the degradation of STING depending on the Beclin-1-mediated autophagy.

### Beclin-1 interacts with the C-terminal region of STING mediating its acetylation

**Fig. 4:**
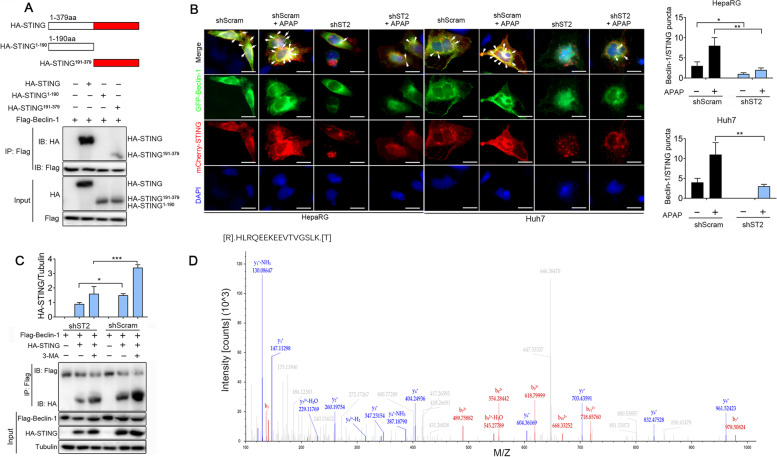
Beclin-1 interacts with the C-terminal region of STING mediating its acetylation. **A** Schematic diagram of STING truncation mutants. HA-STING or its mutants STING^1−190^ and STING.^191−379^ were individually transfected into HepaRG cells along with Flag-Beclin-1. Cell lysates were immunoprecipitated with the anti-Flag antibody and then immunoblotted with the indicated antibodies (*n* = 3). **B** Confocal microscopy of stably shScram or shST2 cells that were transfected with mCherry-tagged STING and GFP-tagged Beclin-1 followed by APAP (10 mmol/L) administration for 12 h (*n* = 3). DAPI stained the nucleus. The right panel showed the colocalization index (number of punctate/cell) between STING and Beclin-1. Scale bars, 10 μm. **C** Cells were co-transfected with HA-STING and Flag-Beclin-1 plasmid for 24 h, and then treated with 3-MA (10 mM) for 12 h. Cell lysates were subjected to co-immunoprecipitation and immunoblotting to analyze the interaction between STING and Beclin-1. The anti-HA anti-Flag antibodies were used. **D** LC–MS/MS analysis of STING identifies acetylated K338. Acetyl peptides corresponding to HLRQEEKEEVTVGSLK containing K338 were identified. The MS/MS spectra for the acetylated peptide HLRQEEKEEVTVGSLK were shown. *P < 0.05, **P < 0.01, ***P < 0.001

We next truncated HA-tagged STING as an N-terminal (aa 1 to 190) truncation mutant or C-terminal (aa 191 to 379) truncation mutant to investigate the regions necessary for the Beclin-1 and STING interaction. In HepaRG cells, HA-STING^191−379^ but not HA-STING^1−190^ protein was precipitated by Beclin-1 (Fig. 4A). Thus, the C-terminal region of STING, but not the N-terminal region, was critical for the interaction between STING and Beclin-1. The mCherry-tagged STING and GFP-tagged Beclin-1 were co-transfected into HepaRG/shST2 cells with or without APAP stimulation. Confocal microscopy observed that the colocalization of STING and Beclin-1 was decreased in HepaRG/shST2 cells with or without APAP treatment (Fig. 4B). To test the effect, if any, of knockdown of ST2 regulation on the interaction between Beclin-1 and STING. Co-immunoprecipitation showed that Beclin-1 interacted with STING (Fig. 4C, lanes 4 and 5), and this interaction detectably decreased in shST2 cells compared with shScram cells (Fig. 4C, lanes 2 and 5).

Now that we have confirmed that IL-33/ST2 is associated with autophagy, what is the mechanism by which IL-33/ST2 affects the interaction of STING and Beclin-1? We purified the HA-STING protein precipitated by Beclin-1 from rhIL-33-stimulated cells and subjected it to LC–MS/MS analysis. We identified a lysine peptide (HLRQEEKEEVTVGSLK) that was mapped to a region of STING containing K338 (Fig. 4D), indicating that STING can be acetylated at the sites of Lys338. These results might indicate that Beclin-1-mediated acetylation of STING at Lys338 inhibited the subsequent STING signal transduction, resulting in the fusion of STING protein with autophagolysosomes.

### IL-33/ST2 disrupts STING/TBK1/IRF3 signal transduction via the autophagy pathway

**Fig. 5:**
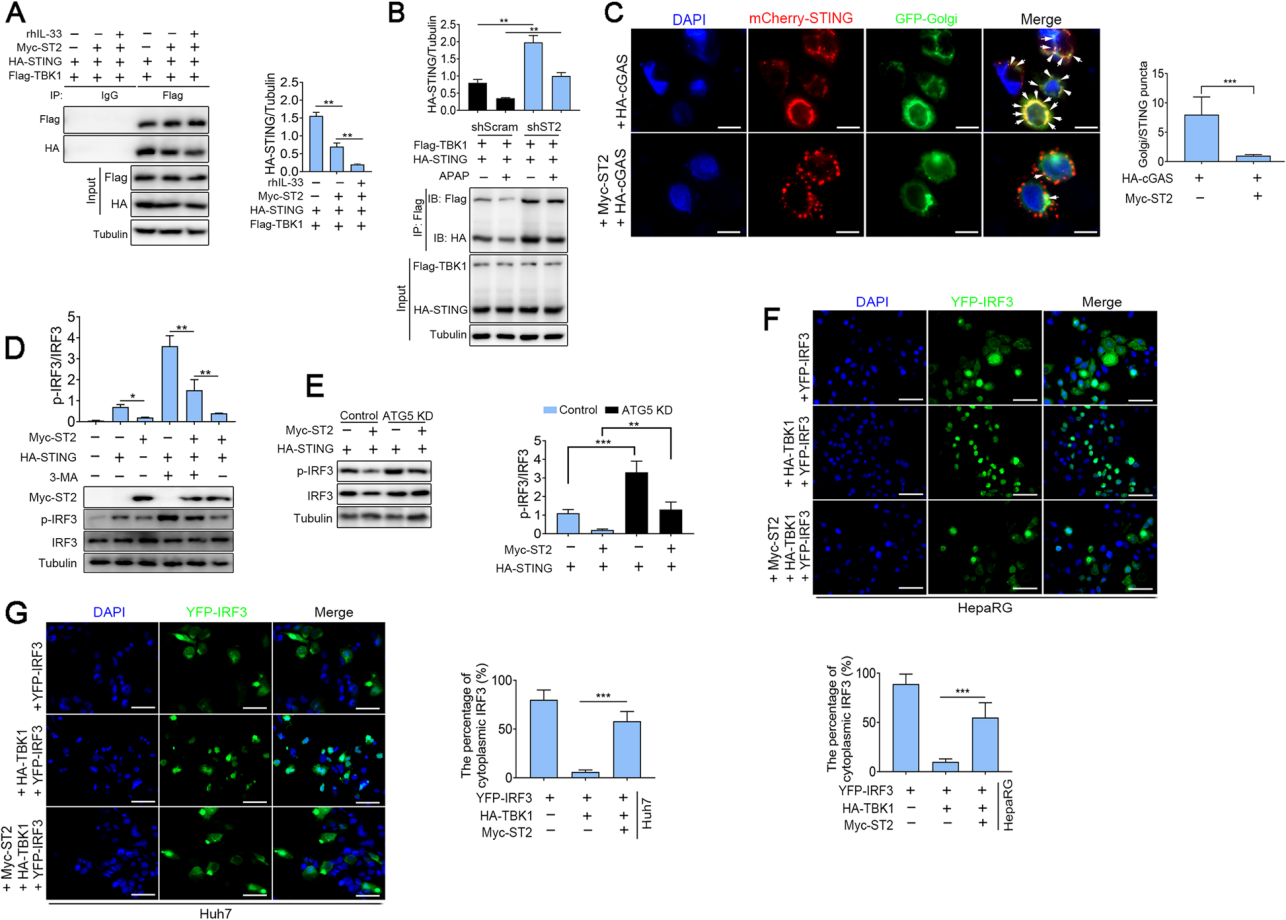
IL-33/ST2 disrupts STING/TBK1/IRF3 signal transduction via the autophagy pathway. **A** Co-immunoprecipitation and immunoblot analysis of lysates of HepaRG cells transfected with plasmids encoding Myc-tagged ST2, HA-tagged STING and Flag-tagged TBK1 without or with rhIL-33 treatment at a final concentration of 100 ng/mL (*n* = 3). **B** The shST2 and shScram cells were co-transfected with HA-STING and Flag-TBK1 plasmid for 24 h and treated with APAP (10 mmol/L) for 12 h. Cell lysates were subjected to immunoprecipitation and immunoblot analysis of the interaction between TBK1 and STING. The anti-HA anti-Flag antibodies were used (*n* = 3). **C** Confocal microscopy of HepaRG cells transfected for 24 h with Myc-tagged ST2, HA-tagged cGAS, mCherry-tagged STING and GFP-tagged Golgi (*n* = 3). DAPI stained the nucleus. The right panel showed the colocalization index (number of punctate/cell) between STING and Golgi, scale bar = 10 µm. **D** HepaRG cells were transfected with plasmids encoding Myc-tagged ST2 and HA-tagged STING for 24 h and then treated with 3-MA (10 mM) for the indicated times. The total cell lysates were immunoblotted with the indicated antibodies (*n* = 3). **E** Immunoblot analysis of IRF3 phosphorylation levels in ATG5 KD cells transfected with Myc-tagged ST2 and HA-tagged STING. **F** and **G** HepaRG and Huh7 cells were transfected with plasmids encoding Myc-tagged ST2, HA-tagged TBK1 and YFP-tagged IRF3 for 24 h. The subcellular localization of IRF3 was visualized by confocal microscopy (*n* = 3), bar = 50 µm. *P < 0.05, **P < 0.01, ***P < 0.001

**Fig. 6:**
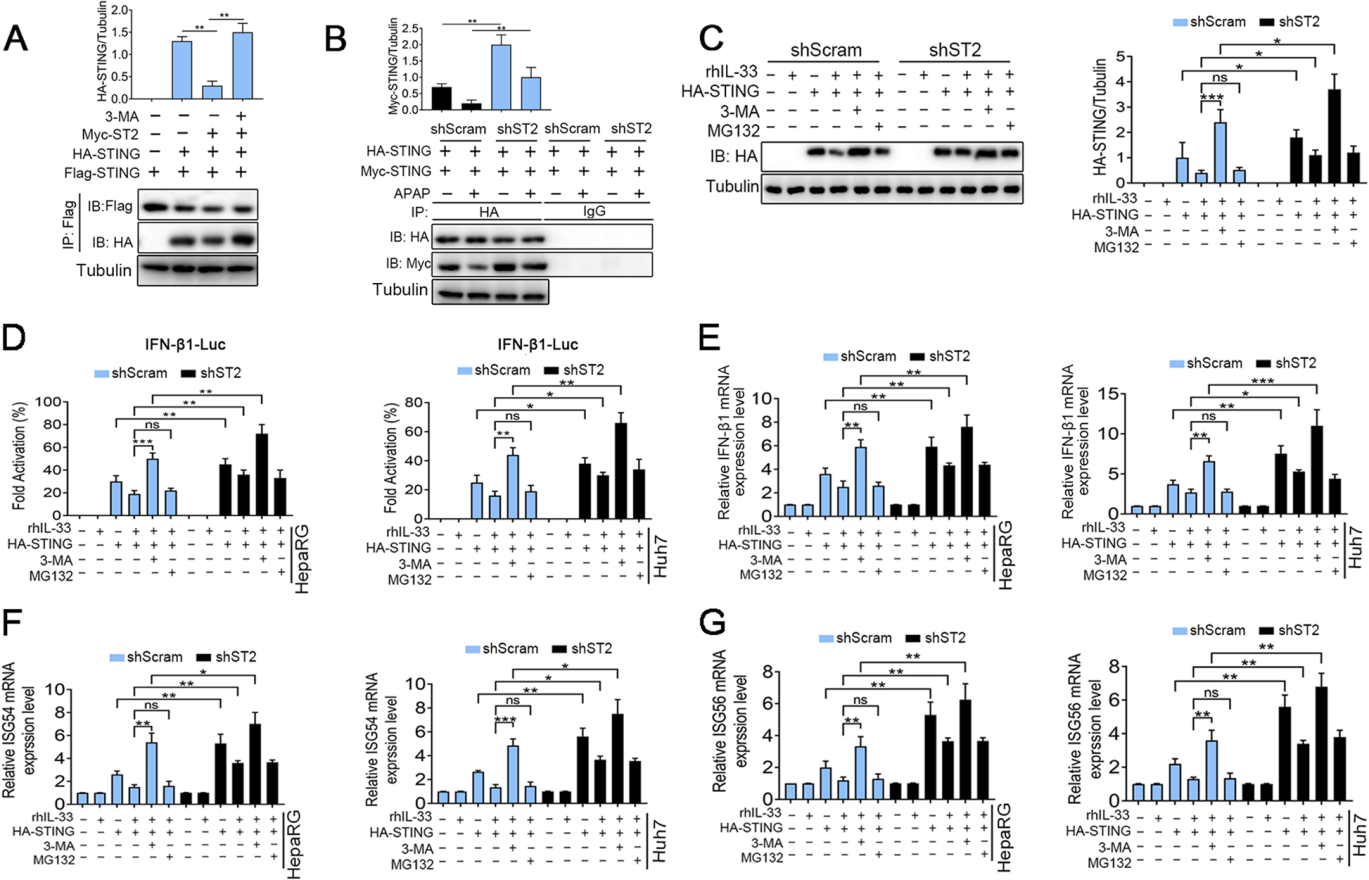
IL-33/ST2 regulates the activity of the IFN-β1 promoter. **A** For immunoprecipitation, anti-HA anti-Flag or anti-Myc antibodies were used (*n* = 3). **B** Co-immunoprecipitation and immunoblot analysis of shST2 and shScram cells co-transfected with HA-STING and Myc-STING plasmid for 24 h treated with APAP (10 mmol/L) for 12 h (*n* = 3). **C** Cells transfected with HA-tagged STING were subjected to pre-incubation with or without rhIL-33 (100 ng/mL) for 2 h and then treated with 10 mM 3-MA for the indicated times. The total cell lysates were then analyzed by western blot with the indicated antibodies (*n* = 3). **D** Luciferase activity was determined and normalized using the dual-luciferase reporter system (*n* = 4). **E**—**G** The qRT-PCR was used to test IFN-β1, ISG54 and ISG56 mRNA levels in ST2 wild-type and knockout cells. *P < 0.05, **P < 0.01, ***P < 0.001, ns indicates not significant

STING is transported from the endoplasmic reticulum (ER) to the Golgi apparatus, recruiting TBK1 and then activating IRF3 [19]. Subsequently, phosphorylated IRF3 is further dimerized and translocated into the nucleus, leading to transcription of the gene encoding IFN [20]. We already confirmed that IL-33/ST2 promoted the degradation of STING. An important question that needs to be answered was how IL-33/ST2 regulated STING signal transduction. Thus, we explored the effect of IL-33/ST2 on the interaction between TBK1 and STING. The Myc-tagged ST2, HA-tagged STING and Flag-tagged TBK1 were co-transfected into HepaRG cells with or without rhIL-33 treatment for a co-immunoprecipitation assay. From Fig. 5A, lanes 4–6, we could see that exogenous overexpression of ST2 significantly reduced the STING protein levels precipitated by TBK1. In contrast, knockdown of ST2 enhanced STING precipitated by TBK1 under APAP treatment (Fig. 5B, lanes 2 and 4). It is noteworthy that the oligomerization of STING and its translocation from ER to a perinuclear compartment were dampened by adding ST2 (Fig. 5C). Then, we assessed the phosphorylation of IRF3. The results showed that exogenous overexpression of ST2 could significantly inhibit the STING-dependent phosphorylation of IRF3 (Fig. 5D). With or without exogenous high expression of ST2, knockdown of ATG5 significantly increased the phosphorylation level of IRF3 (Fig. 5E). We also found that the translocation of IRF3 into the nucleus was significant under exogenous expression of TBK1 in HepaRG and Huh7 cells, whereas over-expression of ST2 decreased the effects (Fig. 5F and G). Next, we explored the inhibitory effect of ST2 on the formation of STING dimerization. Surprisingly, exogenous ST2 significantly inhibited STING dimer formation (Fig. 6A, lanes 2 and 3), whereas 3-MA treatment reversed this effect (Fig. 6A, lanes 3 and 4). Conversely, silencing ST2 enhanced the STING dimerization (Fig. 6B, lanes 2 and 4).

### IL-33/ST2 inhibits the activity of the IFN-β1 promoter

Activated STING recruitment TBK1 promotes the phosphorylation of STING, which in turn recruits IRF3 [21]. Phosphorylated IRF3 dimerization into the nucleus, binding to the IFN promoter region, induces IFN-β1 transcription [22]. To further explore the relationship between IL-33/ST2 and STING/IFN signal, we generated constructs with the IFN-β1 promoter region in the pGL4-enhancer vector and performed a luciferase activity assay. The HA-tagged STING, pGL4-IFN-β1 promoter-Luc reporter, or pGL4-basic control vectors were co-transfected into HepaRG cells, respectively, treated with 3-MA or MG132. Immunoblotting analysis validated that the expression of HA-tagged STING protein was increased in shST2 cells compared with shScram group, which was aggravated by 3-MA treatment, but not by MG132 treatment (Fig. 6C). The results of the luciferase assay demonstrated that 3-MA treatment increased the luciferase activity of the IFN-β1 gene promoter by ~ 30% in shScram cells treated with rhIL-33 stimulation. Knocking down ST2 increased the activity of the IFN-β1 gene promoter compared with the shScram cells after rhIL-33 stimulation, this effect was promoted by 3-MA treatment, but not by MG132 treatment (Fig. 6D). These findings confirmed that IL-33/ST2 may have a negative regulatory effect on the transcription of the IFN gene. Thus, the potential regulatory effect of IL-33/ST2 on STING/IFN was further explored. Next, we examined the effects of IL-33/ST2 on the mRNA level of IFN-β1, and its downstream factors ISG54 and ISG56, also named IFN-induced proteins with tetratricopeptide repeats (IFIT) 1 and IFIT2. The relative mRNA expression of IFN-β1, ISG54 and ISG56 was significantly increased in HepaRG and Huh7 cells co-transfected with HA-tagged STING after stimulation with 3-MA (Fig. 6E-6G).

### Blocking IL-33/ST2 promotes IRF3 translocation upon APAP stimulation

**Fig. 7:**
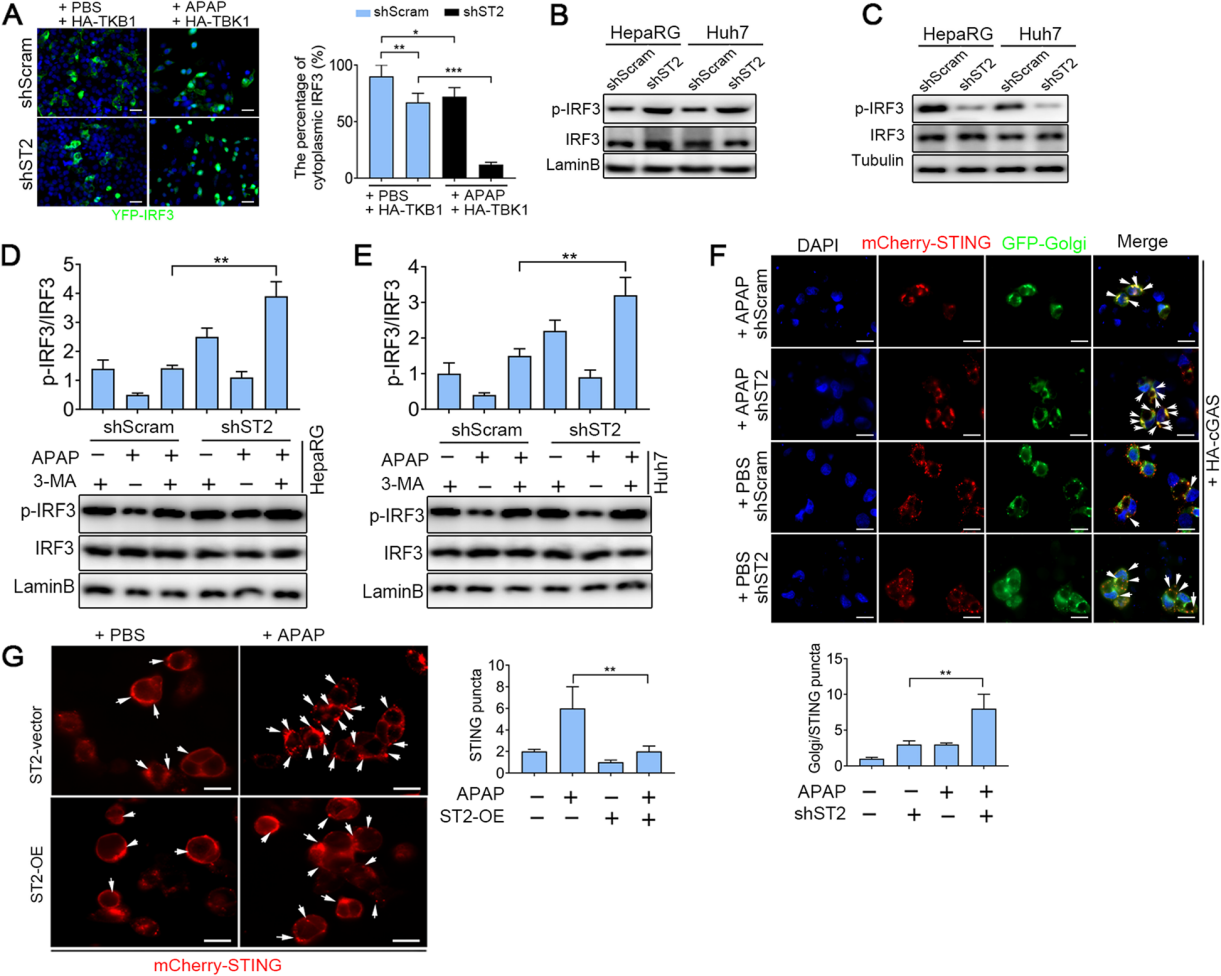
Blocking IL-33/ST2 promotes STING signal transduction upon APAP stimulation in HepaRG and Huh7 cells. **A** HepaRG/shST2 and HepaRG/shScram cells were co-transfected with HA-TBK1 and YFP-IRF3 plasmid for 24 h and then treated with APAP (10 mmol/L) for 12 h. The accumulation of nuclear translocation of IRF3 and cytoplasmic IRF3 was conducted by confocal microscopy analysis (*n* = 3), bar = 50 µm. **B** and **C** The level of IRF3 phosphorylation in the nucleus and cytoplasm was detected by western blot (*n* = 3). **D** and **E** Cells were treated with APAP (10 mmol/L) for 12 h with or without 3-MA (10 mM) for 12 h. The level of IRF3 phosphorylation was detected by western blot. **F** HepaRG/shST2 and HepaRG/shScram cells were co-transfected with Golgi-tagged GFP, mCherry-tagged STING and HA-tagged cGAS. After 24 h, the distribution of STING was observed by confocal microscopy (*n* = 3). DAPI stained the nucleus. The under panel showed the colocalization index (number of punctate/cell) between STING and Golgi, bar = 10 µm. **G** HepaRG/ST2-Vector and HepaRG/ST2-OE cells were co-transfected with mCherry-STING, followed by APAP (10 mmol/L) for 12 h. Then the distribution of STING was observed by confocal microscopy. The right panel showed the colocalization index (number of punctate/cell), bar = 10 µm. *P < 0.05, **P < 0.01, ***P < 0.001

To better verify the relationship between IL-33/ST2 and STING/TBK1/IRF3 signaling in the presence of APAP stimulation, the YFP-tagged IRF3 and HA-tagged TBK1 were co-transfected into HepaRG/shScram and HepaRG/shST2 cells treated with APAP. Confocal microscopy analysis suggested that the cytoplasmic IRF3 was reduced in HepaRG/shST2 cells. A similar trend was also seen in the presence of APAP stimulation (Fig. 7A). By extracting nuclear and cytoplasmic cellular fractions, we found that the level of IRF3 phosphorylation in the nuclei of ST2 knockdown cells was significantly higher than that of shScram cells, while the IRF3 phosphorylation level of cytoplasm was the opposite (Fig. 7B and C). Additionally, compared with the shScram group, the level of IRF3 phosphorylation in the nuclei was markedly augmented in ST2 knockdown cells subjected to pre-incubation with APAP and then treated with 3-MA (Fig. 7D and E, lanes 3 and 6). Next, we used GFP-tagged Golgi, mCherry-tagged STING and HA-tagged cGAS plasmids and found that the aggregative distribution of STING in the Golgi apparatus was enhanced in HepaRG/shST2 cells (Fig. 7F). These results suggested that IL-33/ST2 promoted IRF3 translocation by affecting autophagy.

As the knockdown of ST2 affected STING signal transduction, we further evaluated the transport and activation of STING in ST2-Vector and ST2-OE cells transfected with mCherry-STING upon APAP treatment. We found that the STING translocation from ER to a perinuclear compartment was increased in the presence of APAP stimulation. On the contrary, after the overexpression of ST2, this transformation was reversed (Fig. 7G). Together, these results indicated that IL-33/ST2 signaling inhibited APAP-induced STING signaling activation.

### Recombinant IL-33 and cGAS/STING inhibitor combination therapy delay APAP-induced acute liver injury

**Fig. 8:**
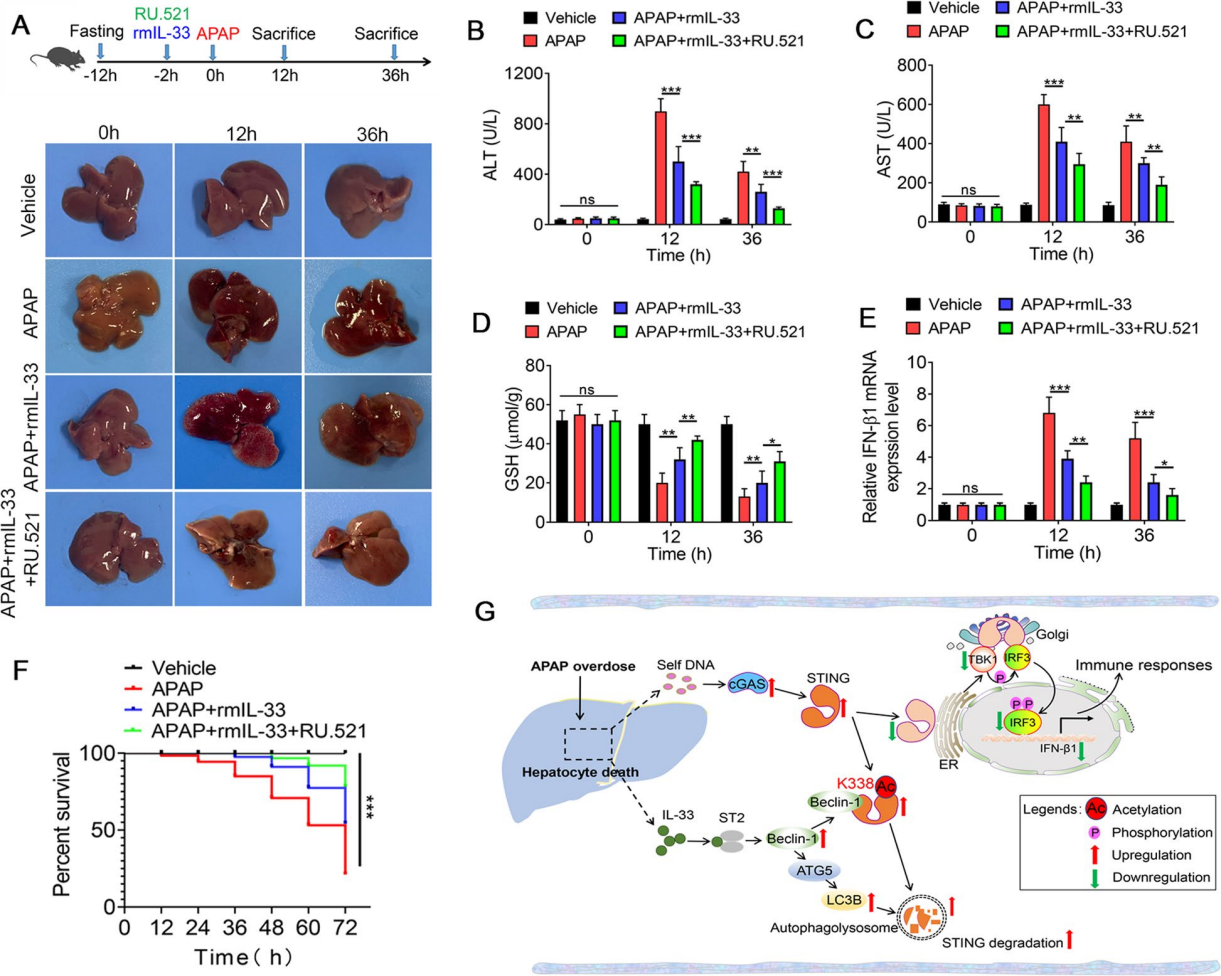
Recombinant IL-33 combines with cGAS/STING inhibitor delaying APAP-induced acute liver injury. **A** Experimental setup illustrating the regimen and timeline. Representative images were used to observe hepatic necrosis in WT mice at 0 h, 12 h and 36 h after APAP (250 mg/kg) injection with or without cGAS/STING inhibitor RU.521 (200 μg/kg) and recombinant IL-33 (500 ng/mouse) (1% DMSO diluted with corn oil as a vehicle) (*n* = 6). **B**—**D** Serum ALT/AST and liver GSH levels were detected (*n* = 6). **E** The mRNA levels of IFN-β1were measured by qRT-PCR (*n* = 6). **F** Survival curve of mice in response to APAP with or without RU.521 and recombinant IL-33 (*n* = 20). *P < 0.05, **P < 0.01, ***P < 0.001, ns indicates not significant. **G** Diagram of the proposed mechanism by which IL-33/ST2 regulates APAP-induced STING signaling via Beclin-1 mediated autophagy. APAP induced massive DNA accumulation and type 1 IFN production accompanied by IL-33/ST2 signaling activation. IL-33/ST2 signaling augmented the interaction between Beclin-1 and STING, resulting in disrupting the dimerization of STING, the phosphorylation of IRF3 and subsequent nuclear transport as well as the transcription of IFN-β1. Beclin-1 binding to the C-terminal region of STING mediated its Lys338 acetylation, leading to autophagy degradation of STING. Moreover, the combination of recombinant IL-33 and cGAS/STING inhibitor RU.521 treatment significantly recovered AILI

Having tested the important relationship between IL-33/ST2 and STING signal during APAP stimulation, we then sought to evaluate the therapeutic potential of cGAS/STING inhibition in APAP-induced liver injury. The cGAS/STING inhibitor RU.521 [23, 24] combined with rmIL-33 was administered 2 h before APAP overdose. The schematic diagram of the work model was exhibited (Fig. 8A). The pathological images of mouse liver injury were assessed at 0 h, 12 h and 36 h after APAP treatment (Fig. 8A). Compared with recombinant IL-33 alone, recombinant IL-33 together with RU.521 significantly repressed APAP-induced liver injury, as demonstrated by greatly decreased ALT/AST levels (Fig. 8B and C) and increased GSH levels (Fig. 8D). More importantly, combing recombinant IL-33 and RU.521 significantly inhibited the APAP-induced mRNA level of IFN-β1 and greatly improved survival in WT mice following a dose (250 mg/kg, i.p.) of APAP treatment (Fig. 8E and F). These findings implied the therapeutic potential of targeting STING signals for the treatment of APAP overdose. Together, we proposed a working model to illustrate how IL-33/ST2 signaling negatively regulates STING signaling during APAP treatment (Fig. 8G).

## Discussion

Although APAP is a popular analgesic, an overdose results in severe centrilobular hepatocyte necrosis [25]. The treatment strategy for drug-induced liver injury is still in its early stages, owing to our lack of understanding of its pathogenesis. The inflammatory response induced by APAP overdose was known to be the primary driver of APAP-induced hepatotoxicity progression [26]. Both pathological and physiological conditions can lead to self-DNA trafficking. Numerous innate immune DNA-sensing pathways activate an antimicrobial type 1 interferon response in response to DNA sensing [27]. An excessive amount of APAP causes DNA fragments, which are known to produce the second messenger cGAMP and cause a type 2 immunological reaction. Abnormal cytoplasmic DNA accumulation was observed in hepatocytes after toxicity in vitro and progressive deposition of liver DNA in vivo [15].

A study revealed that they employed APAP at a dosage of 600 mg/kg, which is near the lethal dose of 750 mg/kg, and that this caused liver non-parenchymal cells to produce more DNA sensors than hepatocytes do during homeostasis [28]. However, we noted that the majority of hepatocytes displayed DNA sensor accumulation after toxic cell injury following treatment with 250 mg/kg APAP. DNA was released from necrotic hepatocytes and non-parenchymal liver cells, amplifying the liver injury. Because hepatocyte necrosis resulted in the release of DAMPS such as HMGB1 and DNA fragments after APAP overdose [4]. Therefore, the toxicological immune response may be triggered to varying degrees at different APAP doses. According to our hypothesis, low-dose APAP stimulation causes hepatocytes to react most favorably, which causes a variety of inflammatory and innate immunological reactions to be triggered.

IL-33, a member of the IL-1 family of cytokines, defined as an “alarm protein” released from necrotic cells, exerts diverse functions through signaling via its receptor ST2. Emerging studies have reported that IL-33/ST2 plays a critical role in the process of different liver diseases [29, 30]. In addition, studies have shown that IL-33 could inhibit ST2/PI3K/mTOR mediated autophagy in allergic rhinitis [31]; It also provided neuroprotection by inhibiting autophagy [32]. However, its role in APAP-induced liver injury has not been fully elucidated. Massive DNA accumulation can trigger different DNA sensors including cGAS, STING and TLR9, leading to STING-mediated activation of IRF3 and production of type I IFNs [21, 33]. Here, we expanded our previous findings that IL-33 was involved in APAP-induced liver necrosis. This work unveiled that IL-33-deficient mice aggravated APAP-induced hepatotoxicity, massive DNA accumulation and type 1 IFN production.

Recent studies have shown that blocking STING/TBK1 signaling plays a central role in many diseases such as lung adenocarcinoma [34], chronic pain and lupus [35, 36]. In addition, other studies have demonstrated the important relationship between IL-33/ST2 and STING/IFN signaling. STING activation in alveolar macrophages and group 2 innate lymphoid cells suppressed type 2 lung immunopathology and airway hyperreactivity induced by IL-33 [37]. Furthermore, IL-33 and its receptor, ST2, are required for cGAMP-induced allergic inflammation in the lung, which may be novel therapeutic targets for allergic asthma [38]. In this study, we further supplemented the new evidence of the relationship between IL-33/ST2 and STING/IFN signaling. We confirmed that IL-33/ST2 potentiated the interaction between Beclin-1 and STING. The more in-depth analysis demonstrated that the C-terminal region of STING, but not the N-terminal region, was critical for the interaction between STING and Beclin-1.

Post-translational modifications of STING proteins are critical for STING signaling. One study reported that phosphorylation at S366 of STING protein was required for IRF3 activation [39]. However, another study claimed that S366 phosphorylation selectively blocked IRF3 activation [40]. Recently, Wang et al. suggested that the need for EGFR for IRF3 activation was not related to STING S366 phosphorylation [41]. These reports illustrated the conflicting effects of S366 phosphorylation on STING signaling. Our results demonstrated that Lys338 acetylation leads to autophagy degradation of STING. IL-33/ST2 signaling augmented the interaction between Beclin-1 and STING, resulting in disrupting the dimerization of STING, the phosphorylation of IRF3 and subsequent nuclear transport as well as the transcription of IFN-β1.

Marques et al. provided that blockage of DNA recognition by the innate immune system might consist of a promising therapeutic avenue in DILI [15]. Therefore, blocking cGAS/STING signaling may improve AILI. C-176 is a potent and covalent mouse STING inhibitor that has the potential to significantly lower serum levels of type I IFNs by inhibiting STING-mediated IFN reporter activity and reducing IFN reporter activity in general. Though it has a brief serum half-life, it binds to C91 in STING to exert its effects [42]. RU.521 is a selective cGAS inhibitor that represses cGAS-mediated upregulation of interferon. In this work, we combined RU.521 and recombinant IL-33 to treat APAP-induced liver injury. The combination of recombinant IL-33 and RU.521 treatment reduced the APAP-mediated induction of serum levels of ALT/AST and improved survival, compared with recombinant IL-33 alone. Although we showed for the first time that RU.521 significantly repaired APAP-induced acute liver injury in vivo, the effect of long-term treatment with RU.521 needs to be further explored.

## Conclusions

In conclusion, our findings established a pivotal role for IL-33/ST2 in regulating the activation of cGAS/STING in APAP-induced liver injury. Targeting STING signaling may be a novel strategy for the treatment of acetaminophen hepatotoxicity.

## Supplementary Information

**Additional file 1.**

**Additional file 2: Table S1.** The primer sequences used for cDNA amplification.

**Additional file 3:** **Table S2.** The primer sequences used for cDNA real-time RT-PCR.

## Data Availability

The datasets generated during and/or analyzed during the current study are available from the corresponding author on reasonable request.
