# Supplementary figures and images for: IL-33/ST2 antagonizes STING signal transduction via autophagy in response to acetaminophen-mediated toxicological immunity
Source: Cell Commun Signal. 2023 Apr 20;21:80. doi: 10.1186/s12964-023-01114-3 (PMC10116723; doi:10.1186/s12964-023-01114-3)

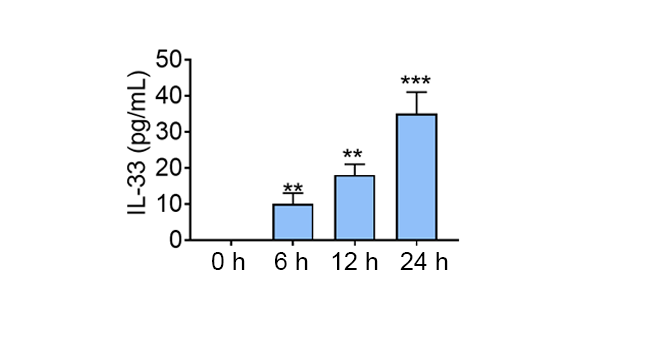

Supplement: Supplementary file 2 — Additional file 1. [file 12964_2023_1114_MOESM1_ESM.tif]
