# Supplementary material for: IL-33/ST2 antagonizes STING signal transduction via autophagy in response to acetaminophen-mediated toxicological immunity
Source: Cell Commun Signal. 2023 Apr 20;21:80. doi: 10.1186/s12964-023-01114-3 (PMC10116723; doi:10.1186/s12964-023-01114-3)
Supplement: Supplementary file 3 — Additional file 2: Table S1. The primer sequences used for cDNA amplification. [file 12964_2023_1114_MOESM2_ESM.docx]

**Table S1** The primer sequences used for cDNA amplification.

|  |  |  |
| --- | --- | --- |
| Plasmids | Restriction enzyme cutting site | Primer (5’– 3’) |
| HA-STING, Flag-STING, Myc-STING, mCherry-STING, | EcoRI, XhoI | CCGGAATTCATGCCCCACTCCAGCCTGCATC (forward) |
|  |  | CCGCTCGAGAGAGAAATCCGTGCGGAGAGGGAG (reverse) |
| HA-TBK1, Flag-TBK1 | BamHI, XhoI | CGCGGATCCATGCAGAGCACTTCTAATCATCTGT (forward) |
|  |  | CCGCTCGAGAAGACAGTCAACGTTGCGAAGG (reverse) |
| HA-cGAS | SacI, XhoI | GGAGCTCATGCAGCCTTGGCACGGAAAGG(forward) |
|  |  | CCGCTCGAGAAATTCATCAAAAACTGGAAACTC(reverse) |
| Flag-Beclin-1, GFP-Beclin-1 | EcoRI, XhoI | CCGGAATTCATGAGTCTGCTAAACTGTGAAAACAGC(forward) |
|  |  | CCGCTCGAGTACCCCTGCATCAGTACTTCGTG(reverse) |
| Myc-ST2, Flag-ST2 | BamHI, XhoI | CGCGGATCCATGGGGTTTTGGATCTTAGCAATT(forward) |
|  |  | CCGCTCGAGCCCTTGGCTGCCCAGAAGCAA(reverse) |
| YFP-IRF3 | EcoRI, XhoI | CCGGAATTCATGGGAACCCCAAAGCCACGGATCforward) |
|  |  | CCGCTCGAGGATTTCCAGGGCCCTGGGGAGAGC(reverse) |
