# Supplementary material for: IL-33/ST2 antagonizes STING signal transduction via autophagy in response to acetaminophen-mediated toxicological immunity
Source: Cell Commun Signal. 2023 Apr 20;21:80. doi: 10.1186/s12964-023-01114-3 (PMC10116723; doi:10.1186/s12964-023-01114-3)
Supplement: Supplementary file 4 — Additional file 3: Table S2. The primer sequences used for cDNA real-time RT-PCR. [file 12964_2023_1114_MOESM3_ESM.docx]

**Table S2** The primer sequences used for cDNA real-time RT-PCR.

| Gene | Primer (5’– 3’) |
| --- | --- |
| mouse GAPDH | F: AGGTCGGTGTGAACGGATTTG |
|  | R: TGTAGACCATGTAGTAGTTGAGGTCA |
| mouse IFN-β | F: AGCTCCAAGAAAGGACGAACA |
|  | R: GCCCTGTAGGTGAGGTTGAT |
| mouse cGAS | F: GAGGCGCGGAAAGTCGTAA |
|  | R: TTGTCCGGTTCCTTCCTGGA |
| mouse STING | F: GGTCACCGCTCCAAATATGTAG |
|  | R: CAGTAGTCCAAGTTCGTGCGA |
| human GAPDH | F: ACACCCACTCCTCCACCTTT |
|  | R: TCTTCCTCTTGTGCTCTTGCT |
| human IFN-β1 | F: ATGACCAACAAGTGTCTCCTCC |
|  | R: GGAATCCAAGCAAGTTGTAGCTC |
| human ISG54 | F: CACATGGGCCGACTCTCAG |
|  | R: CCACACTTTAACCGTGTCCAC |
| human ISG56 | F: GCGCTGGGTATGCGATCTC |
|  | R: CAGCCTGCCTTAGGGGAAG |
